# Unraveling climatic niche evolution: Insights into the geographical distribution of the neotropical social wasp genus *Synoeca* (Hymenoptera, Vespidae, Epiponini)

**DOI:** 10.1371/journal.pone.0306204

**Published:** 2024-06-28

**Authors:** Matheus Cavalcante Viana, Andressa Duran, Rodolpho Santos Telles Menezes

**Affiliations:** 1 Programa de Pós-Graduação em Zoologia, Universidade Estadual de Santa Cruz, Rodovia Jorge Amado, Ilhéus, Brazil; 2 School of Biological Sciences, Monash University, Melbourne, Victoria, Australia; 3 Universidade Estadual de Santa Cruz, Departamento de Ciências Biológicas, Rodovia Jorge Amado, Ilhéus, Brazil; Shiv Nadar University, INDIA

## Abstract

Niche evolution refers to the process by which species undergo changes in ecological interactions, as well as their ability to disperse over time. Our study focuses on the widely distributed neotropical genus of social wasps, *Synoeca* (Hymenoptera, Vespidae, Epiponini). We use ecological niche modeling to investigate the niche evolution of this insects, to explore how species have evolved within and across distinct environmental boundaries, as well as to explore the overlap, equivalence, and similarity between their niches. Our analysis of Predicted Niche Occupancy reveals that species occupy heterogeneous niches in relation to temperature, precipitation, and altitude, similar to the patterns observed in the analysis of the evolutionary history of climate tolerances, which shows that species have evolved to occupy distinct niche ranges. In addition, our niche comparisons indicate that the species do not share similar niches with each other. All these results suggest that Phylogenetic Niche Conservatism may be playing a significant role as a process contributing to the allopatric pattern observed in this genus. This study represents the first investigation of niche evolution in Vespidae, providing valuable insights for future research into the evolutionary dynamics of insects.

## Introduction

The evolution of niches has been extensively researched, leading to significant discoveries regarding the evolutionary processes that shape ecological diversity [1–6]. This phenomenon describes the evolutionary process in which a species undergoes alterations in its ecological interactions, encompassing changes in relationships with both abiotic and biotic factors, as well as its dispersal capacity over time [7]. In this context, dispersal plays a crucial role in niche evolution, allowing species to colonize environments with different abiotic and biotic conditions, exploit previously inaccessible resources, and may result in ecological adaptations, such as seeking new food sources or specializing in habitats [8–10]. Several driving forces, including limited resources, competition, environmental pressures, geographic expansion, and phylogenetic effects, compel species to leave existing environments and colonize new niches [11].

While niches can undergo changes over time, it is expected that phylogenetically related species maintain some degree of niche similarity across their evolutionary history [7,12], a phenomenon referred to as Phylogenetic Niche Conservatism (PNC). This concept suggests that the ecological characteristics of ancestral lineages remain conserved, giving rise to ecological distribution patterns linked to their phylogenetic relationships.

The Neotropical region stands as a significant focal point for scientific inquiry, boasting an extraordinary diversity that encompasses multiple ecosystems, species, and endemism [13]. These biotas are the result of a complex interaction of evolutionary mechanisms, such as vicariance and dispersal, which have acted over geological timescales to shape the current biological diversity [14,15]. Combined with geographical barriers, historical climatic fluctuations, and the region’s environmental heterogeneity, these mechanisms have provided ecological opportunities for different evolutionary lineages to explore diverse niches and undergo diversification [16]. The array of environmental conditions within the neotropical region, intertwined with species interactions within these conditions, has created numerous ecological opportunities for species diversification. This renders the Neotropics a subject for studies elucidating the processes of niche diversification and how species adapt to environmental changes over time [17–21].

*Synoeca* de Saussure, 1852 (Hymenoptera, Vespidae, Epiponini) represents a Neotropical genus of social wasps known for its nest-architecture and swarm-founding behaviour. This genus comprises six distinct species: *Synoeca chalibea* de Saussure, 1852; *Synoeca cyanea* (Fabricius, 1775); *Synoeca ilheensi*s Lopes & Menezes 2017; *Synoeca septentrionalis* Richards, 1978; *Synoeca surinama* (Linnaeus, 1767); and *Synoeca virginea* (Fabricius, 1804). These wasps are characterized by their medium size and can exhibit black, yellow or metallic colors. They are known for constructing nests directly attached to tree branches, a trait that has earned them regional names such as “cachicamas”, “armadillas”, “conchajonas”, “carachupa”, “caba-tatu” or “marimbondo-tatu” in different parts of Latin America [22,23]. These wasps have a wide distribution across the neotropical region, spanning from central Mexico to northern Argentina. However, their distribution patterns show limited geographic overlap. For instance, *S*. *virginea* is exclusively found within the Amazon Forest, while *S*. *chalibea* is also present in the Amazon, but extends its range to Costa Rica and Panama. *Synoeca surinama* is prevalent in South American rainforests and notably in the Brazilian savannah, the Cerrado [24]. *Synoeca septentrionalis* ranges from northwestern South America through Central America and into central Mexico. Lastly, *S*. *cyanea* and *S*. *ilheensis* are restricted to the eastern portion of South America [22,23,25]. This geographic distribution raises questions regarding the ecological and evolutionary drivers behind these distinctions.

In this study, we employ ecological niche modeling techniques to investigate the evolution of ecological niches among *Synoeca* species. We examine the geographical distribution of these species and track changes in their ecological niches over time. Additionally, by exploring niche evolution and phylogenetic conservatism in *Synoeca*, we investigate the historical processes that regulated the geographic distribution of these wasps.

## Material and methods

### Data collection

**Fig 1:**
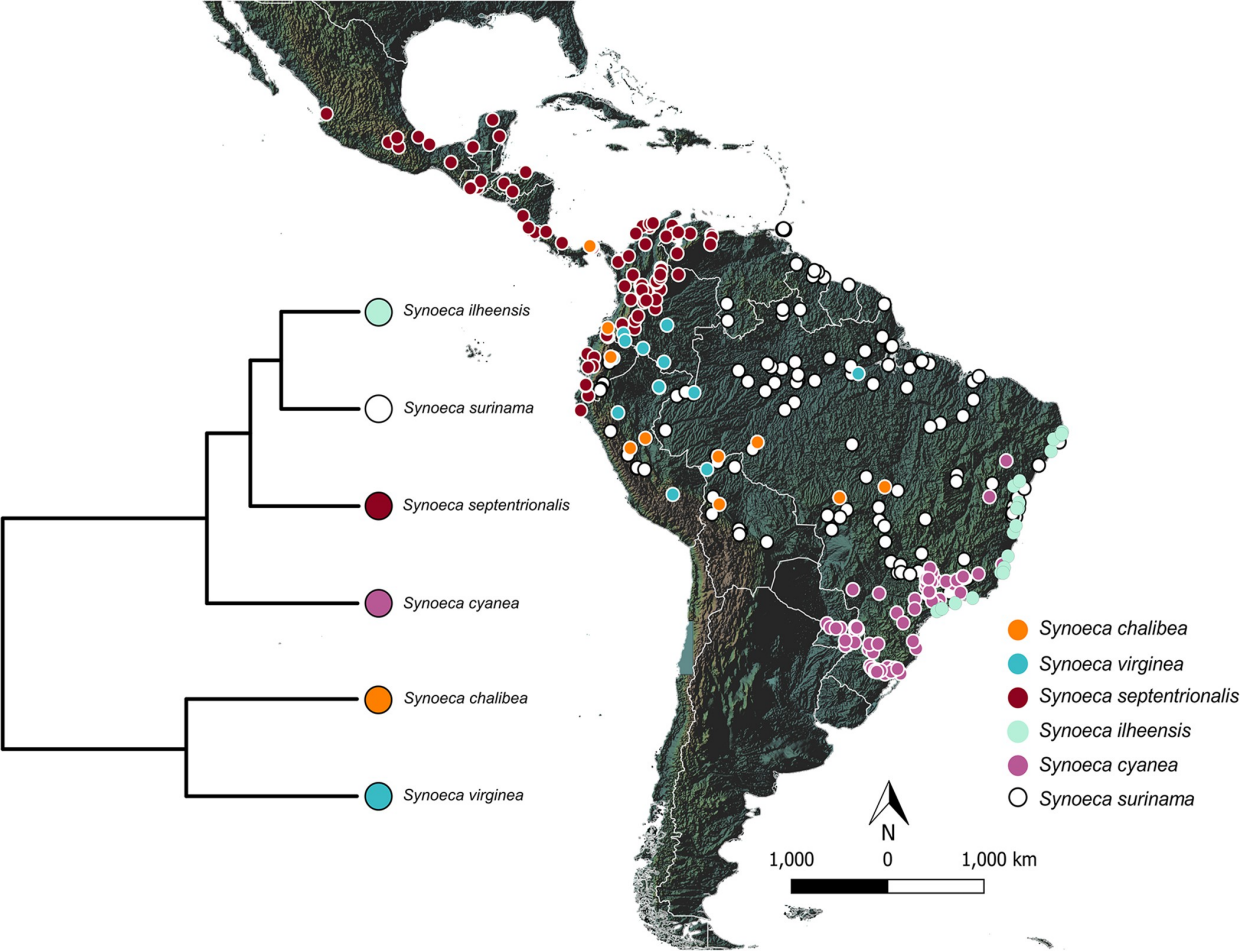
Known occurrence for the six *Synoeca* species. Each circle represents a collection location, distinguished by unique colors corresponding to each species. The color-coded circles align with their respective positions on the phylogenetic tree, as generated by Menezes et al., 2015 [23]. The map was produced using QGIS software version 3.4.4.

To understand the distribution patterns of *Synoeca*, we produced distribution maps for all six species. We used georeferenced occurrence data points (*S*. *chalibea*: 11 points; *S*. *cyanea*: 53 points; *S*. *ilheensis*: 17 points; *S*. *septentrionalis*: 72 points; *S*. *surinama*: 113 points; and *S*. *virginea*: 13 points) **(Fig 1)** obtained through literature and from visits to the museums: American Museum of Natural History (AMNH, New York City), Natural History Museum (NHM, London), Smithsonian National Museum of Natural History (NMNH, Washington, D.C.), and Coleção Entomológica ‘J.M.F. Camargo’ (RPSP) (FFCLRP-USP, Ribeirão Preto, São Paulo, Brazil) (see **S1 Table).** To avoid possible sampling bias, we excluded occurrence data with a distance of less than 25 kilometers for each species [26]. For this purpose, we used the function ’filterByProximity’ from the rangeBuilder [27] package in R 4.1.1 (R Core Team, 2021). For niche modeling and subsequent analyses, we only considered occurrences that had valid coordinates and were within the time range of the available climate variables (1958–2015).

### Construction and selection of climate data

We used current climate variables (1958–2015) of Minimum Temperature, Maximum Temperature, and Precipitation available in the TerraClimate dataset (https://www.climatologylab.org/terraclimate.html) at 2.5 arc-min resolution (≈ 5 km in the tropics) in the construction of the 19 bioclimatic variables using the "biovars" function of the Dismo package [28] implemented in R 4.1.1. We also used the elevation variable obtained through WorldClim v.2 [29]. We applied Pearson correlation test for the variables to eliminate variables with high correlation (≥|0.70|) [30] (see **S2 Table)**. For the construction of the models, the variables selected were: annual mean temperature (BIO01), mean diurnal range (BIO02), isothermality (BIO03), annual precipitation (BIO12), precipitation of driest month (BIO14), precipitation seasonality (BIO15), and elevation.

### MaxEnt modelling

We used the MaxEnt algorithm to build the distribution models [31]. We generated 10 replicates for each species, with 80% of the occurrences to train the model and 20% to test, using the bootstrap method. The performance of this model was evaluated through the realization of the Area under the curve (AUC). The values that comprise this metric range from 0.5 to 1.0, in which, the closer to 1.0, the better the predictions of the potential areas for occurrence of the species. We adopted MaxEnt’s logistic output format to convert the maps to binary using the “Minimum Training Presence Threshold” and later the model was treated in Qgis version 3.4.4.

### Niche occupancy analysis and niche reconstructions

We produced predicted niche occupancy profiles (PNOs) for each of the seven variables employed in the generation of ecological niche models. The PNOs establish correlations between the distribution models (MaxEnt) and the variables, producing graphical representations that depict the predicted niche occupancy of each species in relation to the variables. In addition, we carried out a temporal analysis of the evolution of species’ niches for each phylogenetic niche overlap (PNO) [32]. This analysis estimates ancestral climate tolerances, providing information on how species’ ecological interactions have evolved over time in response to climate change. To achieve this, we used a dated phylogeny for the group generated by Menezes et al. (2015). Both the creation of PNOs and the ancestral niche reconstruction were performed using the ’pno’ and ’anc.clim’ functions, respectively, with the phyloclim [33] package in R 4.1.1.

### Niche comparison analysis

To compare niches among the six *Synoeca* species pairs, we calculated niche overlap metrics using Schoener’s D metric [34]. This metric ranges from 0, indicating complete divergence between species niches, to 1, indicating complete overlap between species niches [6]. Additionally, we conducted hypothesis tests for niche equivalence and similarity. The former examines whether niche overlap remains constant by randomly redistributing population occurrences within their respective ranges. The latter examines whether a population’s niche can predict occurrences more effectively than expected by chance, and vice versa. We performed theses analyses using the ENMTools package [35] in R 4.1.1. Kernel density was employed for all three analyses: niche overlap, niche equivalence, and niche similarity.

## Results

### Suitable habitat in the present-day

**Fig 2:**
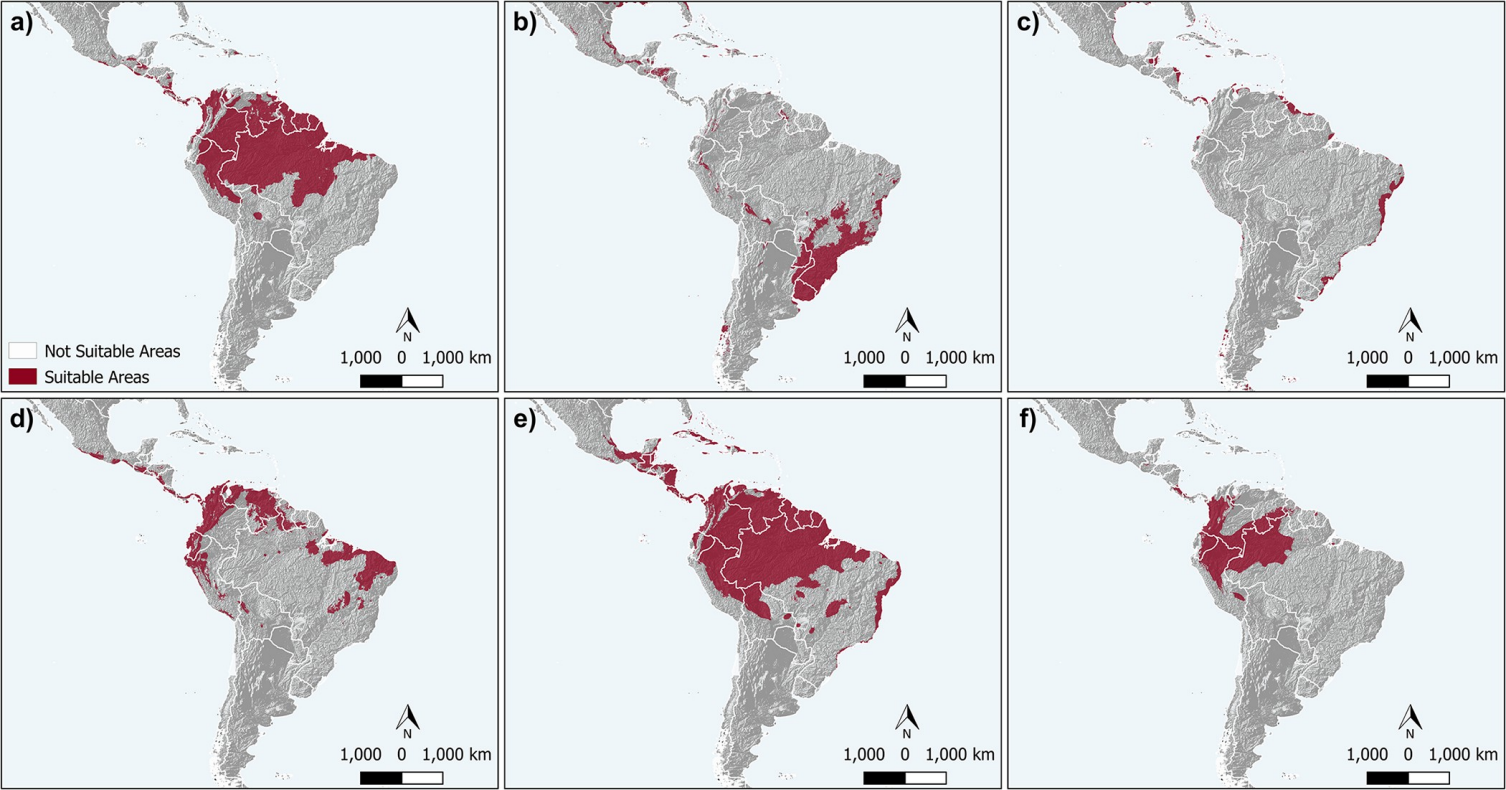
Potential distribution for Synoeca species. In red are the most suitable areas predicted by the models and converted into binaries. a) *Synoeca chalibea*; b) *Synoeca cyanea*; c) *Synoeca ilheensis*; d) *Synoeca septentrionalis*; e) *Synoeca surinama*; and f) *Synoeca virginea*. The map was produced using QGIS software version 3.4.4.

The AUCtest (Area Under the Curve) values ranged from 0.75 to 0.96 S3 Table. Overall, the binary maps showed good agreement with the known areas of occurrence for most species. However, there were exceptions. For *S*. *septentrionalis*, the models indicated a larger potential area of occurrence than the actual known area. Additionally, for *S*. *virginea* and *S*. *surinama*, our models failed to predict some areas where these species are found (Fig 2). The results highlight the geographical range of the species studied, with *S*. *septentrionalis* extending as far as Mexico (Fig 2D), while *S*. *cyanea* occupies an area further south, reaching southern Brazil (Fig 2B). *Synoeca surinama* has the widest distribution, covering a large part of the Amazon region and extending as far as eastern Brazil (Fig 2E). *Synoeca virginea* and *S*. *chalibea* are also present in the Amazon region, while *S*. *ilheensis* is restricted to the Brazilian coast (Fig 2A, 2C and 2F, respectively).

### Ancestral climatic tolerances

**Fig 3:**
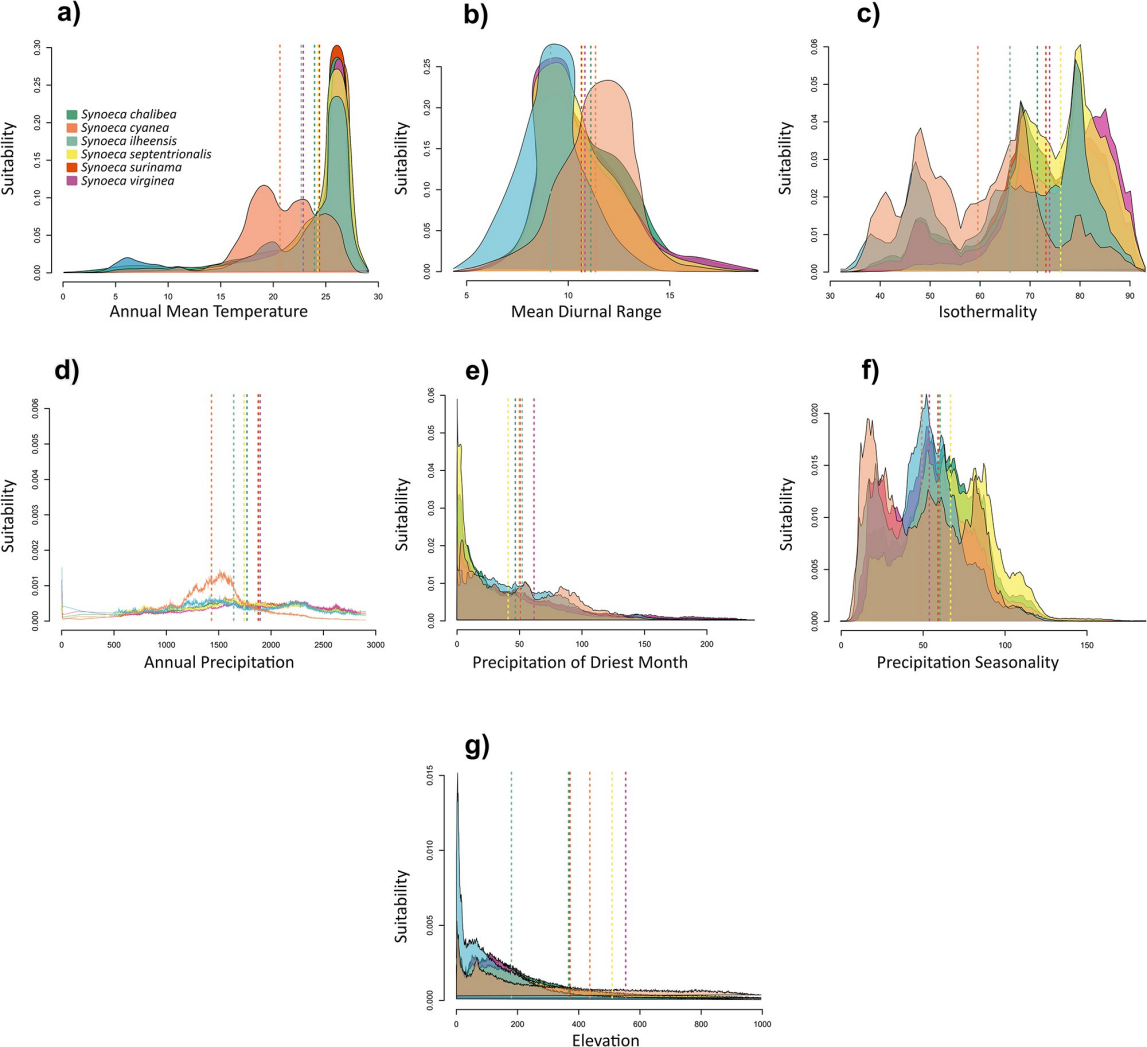
Predicted Niche Occupancy profiles (PNO) for *Synoeca* species. The horizontal axes represent the bioclimatic variables, while the vertical axes indicate the total suitability of each bioclimatic variable index throughout the geographical distribution of each species. The overlapping of peaks in the profiles suggests similar climatic tolerances between the species, while the width of the profile indicates the specificity of the climatic tolerance.

**Fig 4:**
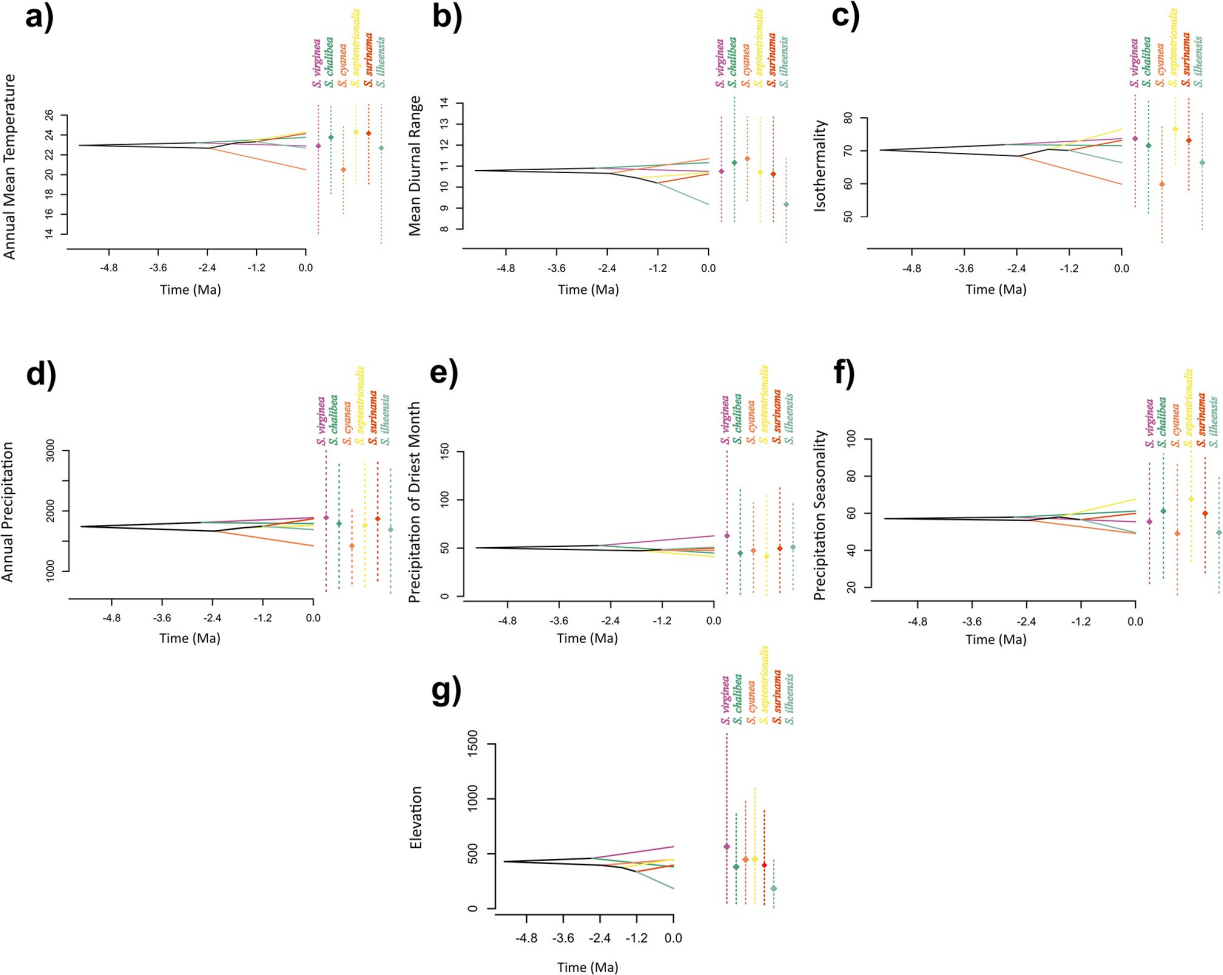
Evolutionary history of climate tolerances in *Synoeca* species. A dated phylogeny of the genus is projected into niche parameter space (y-axis), and the mean climate tolerances, derived from 100 random replications of the predicted niche occupancy profiles are represented at internal nodes. Crossing branches of the phylogenetic tree indicate convergent niche evolution among species, while overlapping internal nodes suggest convergent climatic origins. Vertical dashed lines indicate the 80% central density of climate tolerance for each species, with corresponding points of the same color indicating the mean.

The PNO analyses unveiled heterogeneity in niche occupation, with niche proximity varying depending on the specific variable under consideration. Notably, *S*. *cyanea* and *S*. *ilheensis* exhibited more pronounced differences compared to the other species (Fig 3A–3G). In terms of annual mean temperature, the range spanned from 20°C to 25°C, with *S*. *cyanea* exhibiting a preference for lower temperatures in contrast to the remaining five species (Fig 3A). Our evolutionary history of climate tolerances analysis revealed a declining trend in annual mean temperature for *S*. *cyanea*, while the other species exhibited an inclination towards increasing tolerance to higher temperatures over time (Fig 4A–4G). Concerning mean siurnal range, our PNO results indicated similar tolerances to daily temperature variations (+10°C) for all species, except *S*. *ilheensis*, which displayed lower tolerance compared to the other species (Fig 3B). The niche reconstruction for this variable indicated a general decrease in tolerance over time, with this trend being notably prominent for *S*. *ilheensis* (Fig 4B). Regarding annual precipitation, all *Synoeca* species showed a preference for regions with an average precipitation range of 1400-1800mm (Fig 3D). Niche evolution in this variable remained relatively constant, except for *S*. *cyanea*, which displayed a tendency to endure environments with lower precipitation compared to the other species (Fig 3D). For precipitation in the driest month and precipitation seasonality, all species demonstrated similar occupation profiles, approximately 50mm and over 50mm respectively (Fig 3E and 3F). Elevation proved to be the parameter with the most significant disparities in occupation profiles, with species ranging from just under 200m (*S*. *ilheensis*) to nearly 600m (*S*. *virginea*) (Fig 3G). Niche reconstruction analysis for elevation revealed a general decline in tolerance for higher altitudes over time across most species. Notably, *S*. *virginea* was an exception, as it exhibited an increase in altitude tolerance over time (Fig 4G).

### Niche comparison: Niche overlap, similarity, and equivalence

For our niche overlap analysis, we followed the classification framework proposed by [36], which classifies niche overlap into five levels: 0–0.2 (indicating no overlap), 0.2–0.4 (indicating low overlap), 0.4–0.6 (indicating moderate overlap); 0.6–0.8 (indicating high overlap), and 0.8–1.0 (indicating very high overlap) (Table 1). Our results of niche overlap, along with the PNOs, consistently highlight *S*. *cyanea* and *S*. *ilheensis* as the species with the most distinct niches compared to all others. For these two species, niche overlap values consistently fell within the ’no overlap’ (<0.2) or ’low overlap’ (0.2–0.4) range in all pairwise relationships (Table 1). In contrast, the remaining species exhibited ’moderate overlap’ (0.4–0.6) or ’high overlap’ (0.6–0.8) values in their niche comparisons. In all comparisons between *Synoeca* species, our statistical tests rejected the null hypotheses of equivalence and similarity (P>0.05) (Table 1).

**Table 1:** Measures of overlap (measured by D), equivalence, and niche similarity among Synoeca species. Species abbreviations are as follows: *Synoeca chalibea (S*. *cha)*, *Synoeca cyanea (S*. *cya)*, *Synoeca ilheensis (S*. *ilh)*, *Synoeca septentrionalis (S*. *sep)*, *Synoeca surinama (S*. *sur)*, and *Synoeca virginea (S*. *vir)*.

Comparison a–b	Overlap (D)	Equivalence	Similarity a—b	Similarity b—a
*S*. *cha—S*. *cya*	0.0680300	1	0.5034965	0.4885115
*S*. *cha—S*. *ilh*	0.3534759	1	0.1958042	0.1828172
*S*. *cha—S*. *sep*	0.6020508	0.7326733	0.2377622	0.2477522
*S*. *cha—S*. *sur*	0.7004690	0.1584158	0.0919080	0.0869130
*S*. *cha—S*. *vir*	0.4476905	0.7722772	0.0919080	0.3926074
*S*. *cya—S*. *ilh*	0.0003557	1	0.4745255	0.4725275
*S*. *cya—S*. *sep*	0.2474207	1	0.3196803	0.3186813
*S*. *cya—S*. *sur*	0.1441507	1	0.4255744	0.3866134
*S*. *cya—S*. *vir*	0.0544924	1	0.7342657	0.7262737
*S*. *ilh—S*. *sep*	0.1939805	1	0.3046953	0.3376623
*S*. *ilh—S*. *sur*	0.2617230	1	0.0909090	0.1008991
*S*. *ilh—S*. *vir*	0.2074146	1	0.5004995	0.5184815
*S*. *sep—S*. *sur*	0.5771379	1	0.2697303	0.2827173
*S*. *sep—S*. *vir*	0.4016700	0.9702970	0.4945055	0.2897103
*S*. *sur—S*. *vir*	0.5112963	0.9009901	0.4595405	0.4485514

## Discussion

The potential distribution models generated for the six *Synoeca* species provide important information about the environmental suitability of these insects. Notably, *S*. *surinama* is the species with the most widespread distribution (Fig 1). Carvalho et al. (2021), covering a large portion of the South America and consequently occurring in more diverse environments than all the other *Synoeca* species. Although there is limited overlap in the distribution between the *Synoeca* species, the potential distribution models indicate a more extensive area of overlap than is generally observed, especially for *S*. *chalibea* and *S*. *septentrionalis* (Figs 1, 2A and 2D). This discrepancy suggests that other factors besides climate may be contributing to the allopatric pattern observed in the distribution of these insects, such as geographical barriers and biotic interactions [37].

Geographical barriers, such as mountain ranges, bodies of water, and geological formations, can limit gene flow and species movement, resulting in distinct geographical distributions [38]. For instance, neotectonics and climatic changes during the late Quaternary played pivotal roles in shaping the genetic structure of *S*. *cyanea* and *S*. *ilheensis* [25]. The tropical Andes is a putative barrier for *S*. *septentrionalis* to reach the eastern portion of South America (Figs 1 and 2D). Consequently, the geographic barrier of the Andes may have limited the adaptation of *Synoeca* species, thus facilitating the allopatric pattern observed, resulting in niche conservatism [39]. Moreover, biotic interactions such as competition, predation, mutualism, and parasitism significantly contribute to defining species’ areas of occurrence [40]. These biotic mechanisms, extending beyond climatic conditions, underscore the intricate factors influencing the actual distribution of the species, adding complexity to our understanding of their ecological dynamics.

Phylogenetic Niche Conservatism (PNC) is a concept that is commonly interpreted in two different ways. First, from the perspective of conservatism as a "pattern", we predict greater niche similarity between phylogenetically close lineages compared to more distant lineages [12,39,41,42]. Second, conservatism as a "process" refers to the tendency of lineages to persist within ecological ranges closely aligned with allowable genetic variation in the face of environmental change [12,43]. When faced with rapid environmental fluctuations, lineages exhibit a propensity to migrate to more favorable environments, rather than adapting immediately. Moreover, adaptation to these new environmental conditions would occur at a slower rate than ecological changes [44]. Thus, conservatism would lead species to allopatric separation over time, resulting in niche divergence [12].

Our Profiles Niche Occupancy (PNOs) analysis for *Synoeca* showed a heterogeneous occupancy pattern for the species across examined variables. Surprisingly, the results did not indicate a greater similarity in niches based on phylogenetic proximity, in contrast to the commonly observed niche conservatism "pattern" in various studies [21,45–47]. Notably, this niche differentiation is particularly pronounced in *S*. *cyanea* (Fig 3). When contemplating the adaptive dynamics of these species over time, two plausible scenarios emerge: (1) the species have retained some ancestral characteristics; (2) the species have specialized their ancestral niches in response to specific environmental conditions. In regions marked by instability or successive transformations, such as the Neotropics, any environmental influence may either preserve ancestral niche characteristics or accentuate niche divergence. In the face of diverse ecological conditions in allopatric populations, the closest analog of the ancestral niche may vary for each population [12]. Our analysis of the evolution of the ancestral niche confirms the findings from the Niche Occupancy Profiles, suggesting a unique and divergent evolutionary path in niche characteristics within the genus *Synoeca*, with *S*. *cyanea* showing particularly pronounced differentiation (Fig 4). These results align with the expectations of niche conservatism as an adaptative ‘process’, implying that *S*. *cyanea* may have experienced heightened environmental pressure in its evolutionary habitat.

In our analyses, including PNOs and ancestral niche tolerance analyses, a notable observation emerges: the six *Synoeca* species do not exhibit similar niches based on their phylogenetic proximity (Figs 3 and 4). This deviation from the expectation of niche conservation, a phenomenon not universally observed [48,49] suggests that factors beyond phylogenetic relatedness contribute to niche divergence. The identification of niche divergence among *Synoeca* sister species was larger than expected by chance leads us to infer the involvement of other processes, such as competition [50] and geographical barriers. This interpretation aligns with hypotheses proposed in other studies and remains consistent with our findings [49,51]. Our observations reveal low or moderate niche overlap between species, a phenomenon probably associated with environmental heterogeneity [52]. The diminished niche overlap further underscores the influence of diverse ecological factors shaping the distinctive niches of these *Synoeca* species, highlighting the intricate interplay of environmental conditions in their evolutionary dynamics.

This study presents the first investigation of niche evolution within social wasps. While studies on niche evolution frequently focus on vertebrates and plants, a substantial knowledge gap persists regarding invertebrates, particularly the order Hymenoptera, a highly diverse group. By focusing on *Synoeca* wasps, our objective was to understand how evolutionary processes may have affected these species and, consequently, the observed patterns of niche divergence in our study. Our findings reveal that niche conservatism, operating as a "process" among other mechanisms, played a crucial role in highlighting divergence within this genus. This initial analysis furnishes valuable insights, laying the groundwork for future investigations into the evolutionary dynamics of social wasps.

## Supporting information

S1 Table Sample locations used in the analysis. (XLSX)

S2 Table Pearson’s Correlation Test for all variables used in the analysis. (XLSX)

S3 Table Area Under the Curve (AUC) analysis and the number of occurrences used to test and train the models. (XLSX)
